# miR29a and miR378b Influence CpG-Stimulated Dendritic Cells and Regulate cGAS/STING Pathway

**DOI:** 10.3390/vaccines7040197

**Published:** 2019-11-26

**Authors:** Abid Ullah Shah, Yanan Cao, Naila Siddique, Jian Lin, Qian Yang

**Affiliations:** 1 College of Veterinary medicine, Nanjing Agricultural University, Wei gang 1, Nanjing 210095, China; abidullahshah@yahoo.com (A.U.S.); yncao1994@163.com (Y.C.); 2 National Reference Laboratory for Poultry Diseases, Animal Sciences Institute, National Agricultural Research Center, Islamabad 44000, Pakistan; naila.nrlpd@gmail.com; 3 College of Life Sciences, Nanjing Agricultural University, Wei gang 1, Nanjing 210095, China

**Keywords:** miR-29a, miR-378b, CpG, Dendritic Cells, cGAS/STING

## Abstract

The Cytosine–phosphate–guanosine (CpG) motif, which is specifically recognized intracellularly by dendritic cells (DCs), plays a crucial role in regulating the innate immune response. MicroRNAs (miRNAs) can strongly influence the antigen-presenting ability of DCs. In this study, we examine the action of miRNAs on CpG-stimulated and control DCs, as well as their effect on cyclic guanosine monophosphate-adenosine monophosphate (GMP–AMP) synthase (*cGAS*) and the stimulator of interferon genes (*STING*) signal pathway. Firstly, we selected miRNAs (miR-29a and miR-378b) based on expression in CpG-stimulated mouse bone marrow-derived dendritic cells (BMDCs). Secondly, we investigated the functions of miR-29a and miR-378b on CpG-stimulated and unstimulated BMDCs. The results showed that miR-29a and miR-378b increased expression of both the immunoregulatory DC surface markers (CD86 and CD40) and the immunosuppressive molecule CD273 by DCs. Thirdly, cytokine detection revealed that both miR-29a and miR-378b enhanced interferon-β (IFN-β) expression while suppressing tumor necrosis factor-α (TNF-α) production. Finally, our results suggest that miR-378b can bind TANK-binding kinase binding protein 1 (*TBKBP1*) to activate the cGAS/STING signaling pathway. By contrast, miR-29a targeted interferon regulatory factor 7 (*IRF7*) and promoted the expression of STING. Together, our results provide insight into the molecular mechanism of miRNA induction by CpG to regulate DC function.

## 1. Introduction

Dendritic cells (DCs) are the most important antigen-presenting cells (APCs) [1]. The ability of DCs to present antigens and initiate an immune response forms a link between the innate and the acquired immune system. This process is known as DC maturation, which is characterized by upregulating major histocompatibility complex class II (MHCII) and co-stimulatory molecules (CD80, CD86, and CD40). Unlike co-stimulatory molecules, DCs can be negatively regulated by recognition of co-inhibitory molecules (sialic-acid-binding immunoglobulin-like lectin-G (Siglec-G) and CD273), containing an immunoreceptor tyrosine-based inhibitory motif (ITIM). Siglec-G, a member of the Siglec family, contains ITIM intracellularly [2]. As a member of the immunoglobulin (Ig) superfamily, CD273 also contains ITIM in its cytoplasmic tail [3].

Cytosine–phosphate–guanosine (CpG) was first discovered when a small DNA from *Bacillus* Calmette–Guerin (BCG) was found to have the ability to induce type I interferon [4]. Since then, CpG motifs are widely used as an effective vaccine adjuvant, especially in mucosal immunity. As an important immunostimulatory molecule, CpG can increase the expression of IgA when administrated together with a virus, and it can alleviate the production of IgG [5]. Previous studies showed that CpG can bind to Toll-like receptor 9 (TLR9) and induce immune cells (DCs, B-cells, natural killer cells, and monocytes/macrophages) to initiate the immune response [6,7,8,9]. Recent studies showed that CpG DNA directly activates DCs by increasing the expression of MHCII and co-stimulatory molecules (CD40, CD80, and CD86), as well as cytokine secretion (Interleukin-6, Interleukin-12, tumor necrosis factor-α, and type I Interferon) [10,11]. The intracellular mechanism of CpG-activating DCs is still unclear, and a recent study found that the cyclic guanosine monophosphate-adenosine monophosphate (GMP–AMP) synthase/stimulator of interferon genes (cGAS/STING) pathway was an effective intracellular signal to activate DCs [12]. We hypothesize that CpG might activate the cyclic GMP–AMP synthase (*cGAS*) and stimulator of interferon genes (*STING*) pathway to boost the immune function of DCs.

MicroRNAs (miRNAs) bind to the 3′ untranslated regions (3′UTRs) of their target messenger RNA (mRNA), and negatively regulate their gene expression at the post-transcriptional level or protein synthesis [13]. However, less is known about the influence of miRNAs on control and CpG-stimulated DCs. Numerous studies identified that a large number of miRNAs can be induced by DNA viruses and influence the relevant immune signaling pathways [14,15,16]. miRNAs also influence the DC’s ability to present antigens and secrete cytokines [17]. Therefore, we tried to investigate whether the miRNAs which target the cytosolic DNA sensing pathway can influence the immune response of DCs. Thus, our purpose was to explore the miRNAs involved in the enhancement of CpG-mediated DC cytokine, co-stimulatory, and co-inhibitory molecule production. A previous study identified several differentially expressed microRNAs on DCs after CpG stimulation [18]. In the present study, we extend our search to explore the mechanism via which miRNAs influence the activity of CpG-induced DCs. Our data provide new insight into the underlying nature of miRNAs regulating the immune response of CpG-stimulated bone marrow-derived dendritic cells (BMDCs) in order to optimize potential therapeutic approaches.

## 2. Materials and Methods

### 2.1. Animals and Ethical Statement

Specific pathogen-free (SPF) 4–6 C57BL/6 wild-type mice of 4–6 weeks were used for each experiment (in total, about 60 mice were used). Mice were obtained from the Animal Research Center of Yang Zhou University and kept under pathogenic-free conditions for at least one week before experiment. This study was approved by the Ethical Committee of Animal Experiments (code number SYXK-Su 2011-0036) of the College of Veterinary Medicine, Nanjing Agricultural University. The care and the use of all animals were conducted in a strict environment according to the Animal Research Committee guidelines of the College of Veterinary Medicine, Nanjing Agricultural University.

### 2.2. miRNA Selection and Quantification

**Figure 1:**
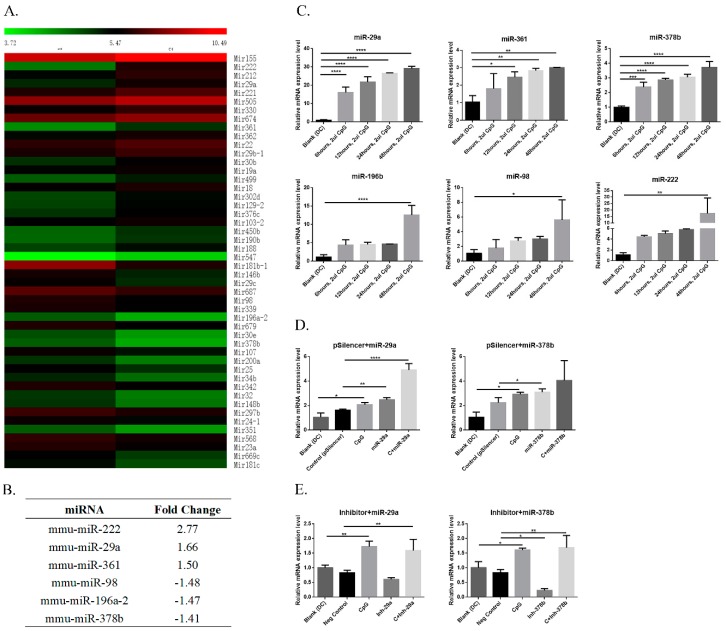
Differential expression of microRNAs (miRNAs) in dendritic cells (DCs) upon cytosine–phosphate–guanosine (CpG) stimulation. (**A**) The heat map of differentially expressed miRNAs on DCs upon CpG stimulation from microarray data. (**B**) List of the candidate miRNAs with their abundance selected after their expression level. (**C**) Result of the qPCR analysis of selected miRNAs (miR-29a, miR-98, miR-196b, miR-222, miR-361, miR-378b) on DCs at different time intervals followed by CpG stimulation. (**D**) qPCR analysis of overexpressed miR-29a and miR-378b, and (**E**) inhibited miR-29a and miR-378b. pSilencer4.1 was used as a negative control, while CpG alone was used as a positive control. All experiments were performed in triplicate. Significant differences between the blank and positive control groups, and negative control and experimental groups are expressed as * *p* < 0.05 or ** *p* < 0.01, *** *p* < 0.001, and **** *p* < 0.0001, respectively. The significance of the data was determined by one-way ANOVA with Dunnett’s multiple comparison test.

The miRNA selection was based on the expression of BMDCs influenced by CpG. Three upregulated and three downregulated miRNAs were selected from our previous microarray data [18] (Figure 1A,B). These miRNAs were isolated from the mouse genome and transfected into mouse BMDCs with X-tremeGENE HP DNA Transfection Reagent from Roche (Mannheim, Germany). Small RNAs were extracted by TRIZOL (Ambion). The miDETECT A Track miRNA qRT-PCR Starter kit purchased from RIBOBIO (Guangzhou, China) was used to perform reverse transcription and qPCR analysis according to the manufacturer’s instructions. 5S rRNA, purchased from RIBOBIO, was used as an internal control to normalize miRNA expression. The primers of six selected miRNAs are listed in Table 1. All experiments were performed in triplicate, and relative expression levels were calculated using the 2^−∆∆Ct^ method [19].

### 2.3. In Silico miRNA Target Site Prediction

Target site prediction of our related miRNAs was based on the CpG DNA sensing pathway, target site conservation, and the thermodynamics of the miRNA–mRNA interaction. We used TargetScan and miRDB software to identify the target sites of our related miRNAs with the 3′UTR of genes encoding cytosolic DNA sensors. Furthermore, to narrow our search, we selected the miRNA–mRNA interactions that were predicted by more than one software (Figure S1, Supplementary Materials).

### 2.4. Cell Isolation, Culture, and Surface Marker Detection

Bone marrow-derived dendritic cells (BMDCs) were isolated from the femurs and tibias of four sacrificed 4–6-week-old wild-type C57BL/6 mice and treated with red-blood-cell lysing buffer (Beyotime) as described previously [20]. Briefly, bone marrow cells were flushed out and cultured in complete medium (Rosewell park memorial institute (RPMI) 1640 with 10% fetal bovine serum (FBS), 1% streptomycin and penicillin, 10 ng/mL recombinant granulocyte-macrophage colony-stimulating factor (GM-CSF) and interleukin-4 (IL-4)) and placed in six-well plates at 37 °C in 5% CO_2_. After six days, the non-adherent immature DCs (1 × 10^6^ cells/mL) were harvested and centrifuged to remove debris and dead cells, and then cultured in complete medium overnight. On the next day, cells were transfected with plasmids, miRNAs, and inhibitors and kept for 48 to 72 h for subsequent assays. All samples were transfected with X-tremeGENE HP DNA Transfection Reagent. Transferred cell samples (1 × 10^6^ cells) were washed twice with phosphate buffered saline (PBS) and incubated at 4 ℃ for 30 min with anti-mouse monoclonal antibodies. The antibodies used to detect surface markers are listed in Section 2.6. Finally, cells were analyzed using a Fluorescence-Activated Cell Sorter (FACS) (BD, FACSVerse) after two separate washes. FACS data were analyzed by FlowJo V10 software (FlowJo, China). At least 10,000 DCs were acquired per sample. All results were expressed as mean fluorescence intensity (MFI). HEK 293T cells were cultured in dulbecco’s modified eagle medium (DMEM) medium containing 10% Lonsa, 100 units/mL penicillin, and 100 g/mL streptomycin at 37 °C in 5% CO_2_, and used for luciferase reporter assays. Cells were transfected with lipofectamine2000 (Invitrogen).

### 2.5. Plasmids and miRNA Inhibitors

The expression vector for miRNAs was constructed based on the pSilencer4.1 vector (Invitrogen). Selected miRNAs were isolated from the mouse genome (we BLAST the sequence of pri-miRNA with the mouse genome, and then selected the flanking region from both sides of the pri-miRNA to design primers), amplified, and then cloned into pSilencer4.1 between the *BamH*I and *Hind*III sites (Figure S2, Supplementary Materials); the primers are listed in Table 2. The 3′UTRs of interferon regulatory factor 7 (*IRF7*) mRNA targets for miR-29a-5p and the 3′UTRs of TANK-binding kinase binding protein 1 (*TBKBP1*) mRNA targets for miR-378b were isolated from the mouse genome, amplified, and cloned into the pMIR-Report luciferase vector (Ambion, TX) between the *Spa*I and *Hind*III sites (Figure S3, Supplementary Materials); the primers are listed in Table 3. Mutations in the mRNA construct were generated by PCR-based site-directed mutagenesis. The sequence of *IRF7* used in the luciferase experiments was changed from 5′–AAATCAG–3′ to 5′–GCTATCA–3′, and the sequence of *TBKBP1* was changed from 5′–AAGTCCAA–3′ to 5′–GGTGTAGG–3′. The miRNA inhibitors were designed and purchased from RIBOBIO (Guangzhou, China). These inhibitors are chemically modified single-chain RNAs, which can be easily obtained and used in miRNA function analysis. Each 100 nM miRNA inhibitor (micrOFFTM mmu-miR-29a-5p inhibitor, micrOFFTM mmu-miR-378b inhibitor, and micrOFFTM inhibitor (negative control)) was transfected with X-tremeGENE HP DNA Transfection Reagent into BMDCs for 2 h, before CpG was added. After another 36–48 h, BMDCs were collected for phenotypic analysis with FACS.

Our study was based on the influence of overexpressed and inhibited miR-29a and miR-378b on unstimulated and CpG-stimulated DCs. Therefore, we divided our experimental samples into two different groups. In the first group, we used the pSilencer4.1 vector to overexpress these miRNAs, while, in the second group, we inhibited these miRNAs and examined their influence on unstimulated and CpG-stimulated DCs. CpG and polyinosinic:polycytidylic acid (Poly I:C) were used directly on DCs as a positive control in each group. Empty pSilencer4.1 and unrelated miRNAs were used as a negative control in the overexpression and inhibition group, respectively.

### 2.6. Reagents and Antibodies

RPMI 1640 and fetal bovine serum were bought from GIBCO (Beijing, China). Recombinant mouse granulocyte-macrophage colony-stimulating factor (GM-CSF) and IL-4 were purchased from Peprotech (Rocky Hill, CT, USA). CpG oligodeoxynucleotides of mouse (1018) phosophorothioated (class-B) [21] at the sites indicated by asterisks (5′–T*G*A*C*T*G*T*G*A*A*C*G*T*T*C*G*A*G*A*T*G*A) were purchased from Novus Biologicals (Centennial, USA). Poly I:C was purchased from Merck (Darmstadt, Germany). X-tremeGENE HP DNA Transfection Reagent was purchased from Roche (Mannheim, Germany). Lipofectamine2000 was obtained from Invitrogen (Shanghai, China). APC-conjugated monoclonal anti-mouse CD11c, PE-conjugated monoclonal anti-mouse CD40 (1C10), PE-conjugated monoclonal anti-mouse CD80 (Clone:16-10A1), PE-conjugated monoclonal anti-mouse CD86 (CloneGL1), APC-conjugated monoclonal anti-mouse MHC (major histocompatibility complex) class II (I-A/I-E) (Clone: M5/114.15.2), APC-conjugated monoclonal anti-mouse CD273 (B7-DC, PD-L2) Clone: TY25, and APC-conjugated monoclonal anti-mouse Siglec-G (Clone: SH2.1) were purchased from eBioscience (San Diego, CA, USA). To detect protein levels, antibodies against the cGAS/STING pathway, *cGAS* rabbit polyclonal (*MB21D1*), *STING* rabbit polyclonal (*TMEM173*), TANK-binding kinase (*TBK1*) rabbit polyclonal, signal transducer and activator of transcription 6 (*STAT6*) rabbit polyclonal (R639), *p-STAT6* rabbit polyclonal (phospho-Y641), c-Jun N-terminal kinase 1/2/3 (*JNK1/2/3*) rabbit polyclonal (P184), *p-JNK1/2/3* rabbit polyclonal (phosphor-T183/Y185), *p38* rabbit polyclonal (T175), *p-p38* rabbit polyclonal (phosphor-Y182), and glyceraldehyde 3-phosphate dehydrogenase (*GAPDH*) rabbit polyclonal were purchased from Bioworld (Nanjing, China), while *IRF3* rabbit monoclonal (EPR2418Y), *IRF7* rabbit monoclonal (EPR4718), and TNF receptor-associated factor 6 (*TRAF6*) rabbit polyclonal were purchased from Abcam (Shanghai, China). Horseradish peroxidase (HRP)-conjugated goat anti-rabbit and goat anti-mouse IgG were also obtained from Bioworld (Nanjing, China). All the antibodies, reagents, chemicals, peptides, recombinant proteins, vectors, inhibitors, commercial assay kits, and software used in this experiment are listed in Table S1, Supplementary Materials.

### 2.7. Quantitative Real-Time Reverse Transcriptase PCR

BMDCs were isolated from four sacrificed 4–6-week-old wild-type C57BL/6 mice, cultured and collected at 24 h or 48 h after treatment with CpG and miR-29a or miR-378b pSilencer4.1 expression plasmid and miRNA inhibitors. Total RNA was extracted with TRIZOL (Ambion) and used to synthesize complementary DNA (cDNA) with the miScript Reverse Transcriptase (Bio-Rad) according to the manufacturer’s protocol. Gene expression was measured by quantitative real-time PCR using gene-specific primers with QuantiTect SYBR Green PCR master mix (Qiagen). Gene expression was normalized to the internal control using GAPDH. Gene-specific primers are listed in Table 4. All experiments were performed in triplicate, and relative expression levels were calculated using the 2^−∆∆Ct^ method [19].

### 2.8. Enzyme-Linked Immunosorbent Assay (ELISA)

The supernatant obtained from the culture of BMDCs was used to perform ELISA of cytokines. Concentrations of TNF-α, IFN-β, C-C motif chemokine ligand 20 (CCL20), and IL-12 in the supernatants were measured using ELISA kits (SenBeiJia Biological Technology Co., Ltd. Nanjing, China) according to the manufacturer’s instructions. The sensitivity of the assay was 25 ng/L for TNF-α, 2 ng/L for IL-12, 3 ng/L for IFN-β, and 5 ng/L for CCL20.

### 2.9. Western Blotting Analysis

BMDCs were isolated from six sacrificed mice and transfected with miRNA pSilencer4.1 expression plasmid and miRNA inhibitors, and then stimulated with CpG and poly I:C at 48 h. Cells were washed twice with ice-cold phosphate-buffered saline (PBS), and lysed with radio immunoprecipitation assay (RIPA) lysis buffer (Biosharp Life Sciences, Beijing, China) and 1% phenyl methyl sulfonyl fluoride (PMSF) protease inhibitor (Solarbio, Beijing, China) for 5 min on ice. Samples were electrophoresed on 10% SDS polyacrylamide gel and transferred to a polyvinylidene difluoride (PVDF) membrane (Biorad, Hercules, CA, USA). After blocking with 5% non-fat milk or 5% bovine serum albumin (BSA) in Tris-buffered saline (TBS) buffer containing 0.05% Tween-20, the membrane was incubated with respective primary antibodies, followed by HRP-conjugated secondary antibodies in the blocking reagent. After extensive washing with TBST, immune reactive bands were analyzed by film exposure after enhanced chemiluminescence (ECL) reaction (Millipore, Bedford, MA, USA). All bands from Western blot were analyzed with Image J software (San Diego, US) to verify the relative expression level.

### 2.10. Dual Luciferase Assay

The predicted target genes were isolated from the mouse genome, and mutations in the mRNAs construct were generated by PCR-based site-directed mutagenesis and cloned into pMIR-Report luciferase vector (Figure S3, Supplementary Materials); the primers are listed in Table 3. To determine the expression of targeted genes with related miRNAs, HEK 293T cells were seeded into a 24-well plate and transfected with 200 ng of various expression plasmids along with 200 ng of pMIR-Report luciferase plasmid; an empty plasmid was used as a control, and 50 ng of pRL-TK plasmid was used as a transfection control. Cells were lysed 48 h post transfection, and a luciferase assay was performed using the dual luciferase reporter assay kit Promega (Madison, WI, USA), according to the manufacturer’s protocol, on a Mudulud Single-tube multimode reader (Promega, Madison, WI, USA).

### 2.11. Statistical Analysis

The results were expressed as means ± SD and analyzed with GraphPad Prism v6.01 (San Diego, US). One-way analysis of variance (ANOVA) was employed to determine significant differences among multiple groups, followed by Tukey’s or Dunnett’s multiple comparison tests. Blank samples containing untransfected DCs were compared with positive controls (CpG and Poly I:C), while all other samples (miR-29a/miR-378b, CpG + miR-29a/miR-378b, inhibitors of miR-29a/miR-378b, and CpG + inhibitors of miR-29a/miR-378b) were compared with control groups (pSilencer4.1 and negative control for miRNA). Differences were considered to be statistically significant when *p* < 0.05. Statistical significance in the figures is indicated as follows: **** p < 0.0001, *** p < 0.001, ** p < 0.01, * p < 0.05; ns, not significant. Data were combined from at least three independent experiments unless otherwise stated.

## 3. Results

### 3.1. CpG Influences miRNAs Level in Bone Marrow-Derived Dendritic Cells (BMDCs)

In the present study, we focused on CpG-stimulated DC maturation and identification of the miRNAs that may influence CpG activity in BMDCs. We selected miRNAs with high and low expression levels in BMDCs after CpG stimulation based on our previous work [18] (Figure 1A). Compared with other studies [22], we found six miRNAs with altered expression in different populations of DCs. Among these commonly selected miRNAs, mmu-miR-29a, mmu-miR-222, and mmu-miR-361 increased, while mmu-miR-98, mmu-miR-196a-2, and mmu-miR-378b decreased in activity in DCs after CpG stimulation (Figure 1B). To confirm the previously obtained microarray data, we examined the expression of six miRNAs listed above, which were stimulated by CpG in mouse BMDCs. qPCR results showed that all miRNAs exhibited peak expression at 48 h, while miR-29a and miR-378b showed continuous increases in significance over various time intervals (Figure 1C). Therefore, we selected miR-29a and miR-378b for further experiments, and we constructed and verified their overexpression vector (Figure 1D) (Figure S2, Supplementary Materials). Moreover, we examined the suppressive effect of an miR-29a and miR-378b inhibitor. The results suggested that miR-29a slightly decreased in expression after inhibition, while miR-378b significantly decreased after inhibition compared to the negative control (Figure 1E). In addition, we found that mir378b was significantly elevated in CpG-stimulated DCs with miR-378b inhibition, but no change was observed in CpG-stimulated DCs with miR-378 overexpression (Figure 1D,E). These results suggest that expression of the selected miRNAs is consistent with our previous microarray data, and that miR-378b influences the activity of CpG on BMDCs.

### 3.2. miR-29a and miR-378b Regulate Co-Stimulatory Molecules of BMDCs

**Figure 2:**
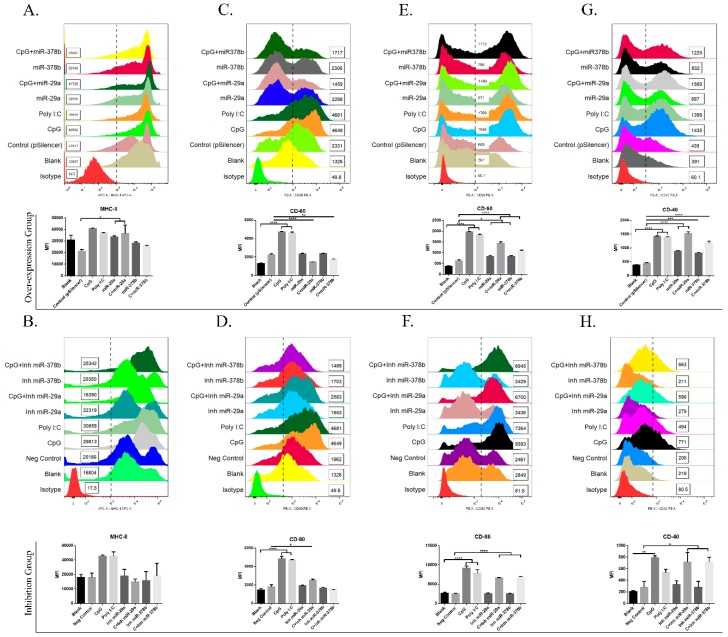
The effect of co-stimulatory molecules on DCs with miR-29a and miR-378b before and after CpG stimulation. (**A**) Flow cytometry analysis of the phenotypic alteration and mean fluorescence intensity (MFI) of major histocompatibility complex class II (MHCII) in overexpression group, (**B**) MHCII in inhibition group, (**C**) CD80 in overexpression group, (**D**) CD80 in inhibition group, (**E**) CD86 in overexpression group, (**F**) CD86 in inhibition group, (**G**) CD40 in overexpression group, and (**H**) CD40 in inhibition group, in bone marrow-derived dendritic cells (BMDCs) influenced by miR-29a and miR-378b with CpG-stimulated and naïve DCs. Blanks containing untransfected DCs and empty vector pSilencer4.1 were used as a negative control; CpG and polyinosinic:polycytidylic acid (Poly I:C) (1 μg/mL) were used as a positive control. All results are representative of three independent experiments. Significant differences between the blank and positive control groups, and negative control and experimental groups are expressed as * *p* < 0.05 or ** *p* < 0.01, *** *p* < 0.001, and **** *p* < 0.0001, respectively. The significance of the data was determined by one-way ANOVA with Tukey’s multiple comparison test.

Consistent with previous research, the present study revealed that miRNAs can regulate the immune responses of DCs [18,20,23,24,25]. In this study, we examined phenotypic changes in CpG-stimulated or unstimulated DCs treated with miR-29a and miR-378b. Fluorescence-activated cell sorting (FACS) results for the surface marker MHCII showed that the mean fluorescence intensity (MFI) of MHCII was enhanced in DCs treated with miR-29a and CpG-stimulated DCs pretreated with overexpressed miR-29a compared to the control group (Figure 2A). In addition, CpG-stimulated DCs preincubated with miR-29a/miR-378b overexpression groups showed significantly reduced expression of CD80 compared with the control group (Figure 2C). Furthermore, the MFI values of CD86 and CD40 increased significantly in the miR-29a/miR-378b and CpG-stimulated miR-29a/miR-378b overexpression groups (Figure 2E,G). Compared with the CpG stimulation group, CpG-stimulated DCs treated with miR-29a/miR-378b inhibitor showed increased MFI for CD40 and CD86 (Figure 2F,H). In addition to the phenotypic alteration of BMDCs, morphological results also indicated that CpG stimulation could accelerate DC maturation. Interestingly, CpG-stimulated DCs preincubated with miR-378b negatively influenced DC maturation compared to those with miR-378b (Figure S4, Supplementary Materials). Together, our results indicate that the selected miRNAs increased co-stimulatory molecules (MHCII, CD40, and CD86) of BMDCs.

### 3.3. miR-29a and miR-378b Modulate the Immunosuppressive Molecules CD273 and Siglec-G in DCs

**Figure 3:**
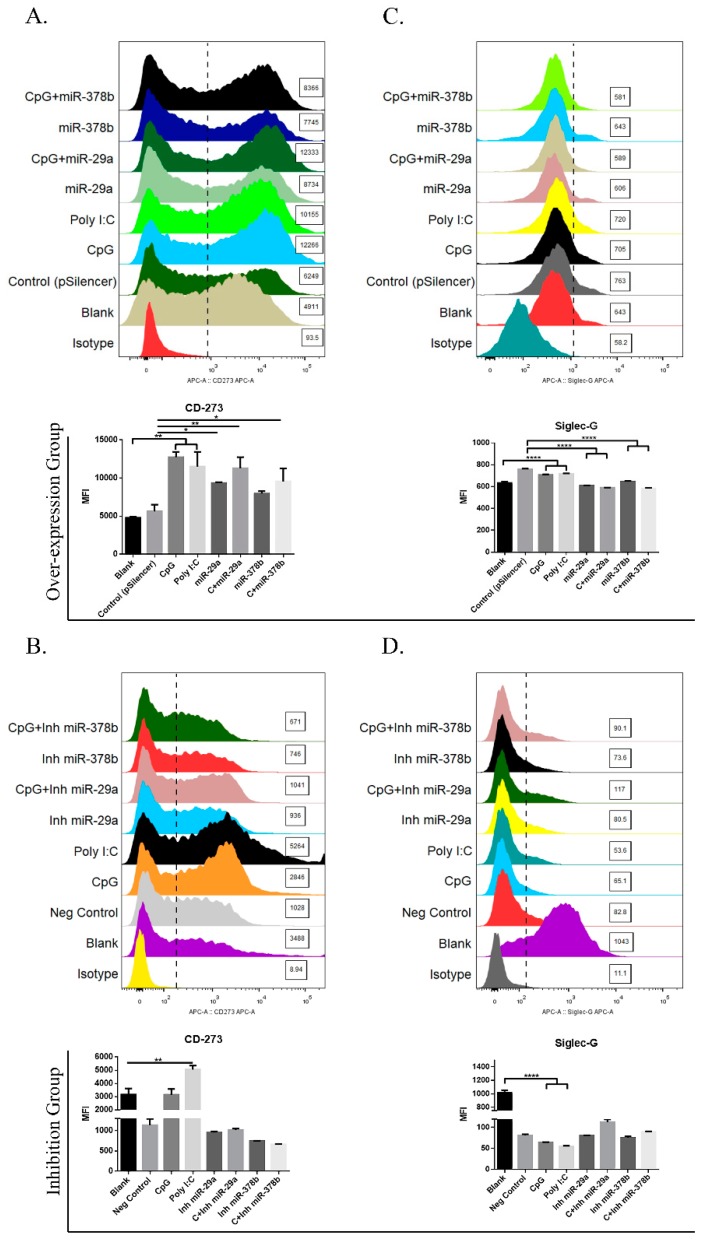
miR-29a and miR-378b regulate expression of immunosuppressive molecules. (**A**) Flow cytometry results of immunosuppressive molecule CD273 in overexpression group, (**B**) CD273 in inhibition group, (**C**) sialic-acid-binding immunoglobulin-like lectin-G (Siglec-G) in overexpression group, and (**D**) Siglec-G in inhibition group in BMDCs. Line 1 shows the overexpression group and line 2 shows the inhibition group of miR-29a and miR-378b with CpG-stimulated and naïve DCs. Blanks containing untransfected DCs and empty vector pSilencer4.1 were used as a negative control; CpG and Poly I:C (1 ug/mL) were used as a positive control. All results are representative of three independent experiments. Significant differences between the blank and positive control groups, and negative control and experimental groups are expressed as * *p* < 0.05 or ** *p* < 0.01 and **** *p* < 0.0001, respectively. The significance of the data was determined by one-way ANOVA with Tukey’s multiple comparison test.

As CD273 and Siglec-G are well-known immunosuppressive molecules in DCs [2,3,26], we examined their phenotypic changes with overexpression or inhibition of miR-29 and miR-378b. The initial results showed that CD273 was significantly upregulated in DCs treated with miR-29a and CpG-stimulated DCs preincubated with miR-29a/miR-378b overexpression groups compared to the control group (Figure 3A). Moreover, Siglec-G was significantly downregulated in DCs treated with miR-29a/miR-378b and in CpG-stimulated DCs pretreated with miR-29a/miR-378b overexpression groups (Figure 3C). Meanwhile, the inhibitor groups did not show any significant changes in CD273 or Siglec-G (Figure 3B,D). These results suggest that miR-29a and miR-378b influence the immunosuppressive molecules of BMDCs.

### 3.4. miR-29a and miR-378b Influence the cGAS/STING Pathway

#### 3.4.1. mRNA Analysis of cGAS/STING Pathway Changes Stimulated by miR-29a and miR-378b

**Figure 4:**
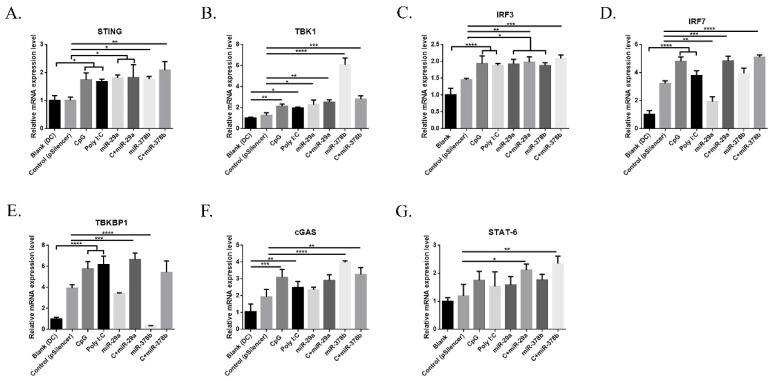
Results of the qPCR analysis following stimulation by overexpressed miR-29a and miR-378b of cyclic guanosine monophosphate-adenosine monophosphate (GMP–AMP) synthase and the stimulator of interferon genes (cGAS/STING) pathway-related genes: (**A**) *STING*, (**B**) TANK-Binding Kinase 1 (*TBK1*), (**C**) Interferon Regulatory Factor 3 (*IRF3*), (**D**) *IRF7*, (**E**) TBK Binding Protein 1 (*TBKBP1*), (**F**) *cGAS*, and (**G**) Signal Transducer and Activator of Transcription 6 (*STAT6*). All these expressions were normalized with Glyceraldehyde 3-Phosphate Dehydrogenase (*GAPDH*) messenger RNA (mRNA) expression level. Empty vectors (pSilencer4.1) were used as a negative control; CpG and Poly I:C were used as a positive control. These results are taken from three independent experiments. Significant differences between the blank and positive control groups and the group treated with pSilencer4.1 are expressed as * *p* < 0.05, ** *p* < 0.01, *** *p* < 0.001, and **** *p* < 0.0001, determined by one-way ANOVA with Tukey’s multiple comparison test.

CpG is recognized by Toll-like receptor 9 (TLR9), a surface receptor that is expressed intracellularly by certain immune cells. The cGAS/STING pathway acts as a cytosolic DNA sensor, inducing type I IFN production [27]. In this study, we firstly investigated mRNA expression of the cGAS/STING pathway in miR-29a and miR-378b overexpression and inhibition groups. Initially, BMDCs treated with miR-29a/miR-378b showed significantly increased mRNA levels of *STING*, *TBK1*, and *IRF3* (Figure 4A–C). Moreover, the mRNA level of *IRF7* was reduced by 40% compared to pSilencer4.1 in the miR29a overexpression group (Figure 4D). miR-378b caused strong downregulation of *TBKBP1* (Figure 4E), but caused upregulation of *cGAS* (Figure 4F). However, the inhibition of miR-29a and miR-378b did not lead to any significant change in the cGAS/STING pathway (Figure S5, Supplementary Materials). CpG-stimulated BMDCs pretreated with miR-29a showed significantly upregulated mRNA levels of *IRF7* compared to the miR-29a group (Figure 4D), indicating that miR-29a might target *IRF7*. Interestingly, we also found that CpG-stimulated DCs preincubated with miR-378b exhibited significantly decreased expression of *TBK1* but increased expression of *TBKBP1* compared to miR-378b (Figure 4B,E). This finding indicates that miR-378b might bind with *TBKBP1* to block *TBK1* function, thereby influencing the cGAS/STING pathway in DCs.

#### 3.4.2. cGAS/STING Pathway Regulatory Proteins Influenced by miR-29a and miR-378b

**Figure 5:**
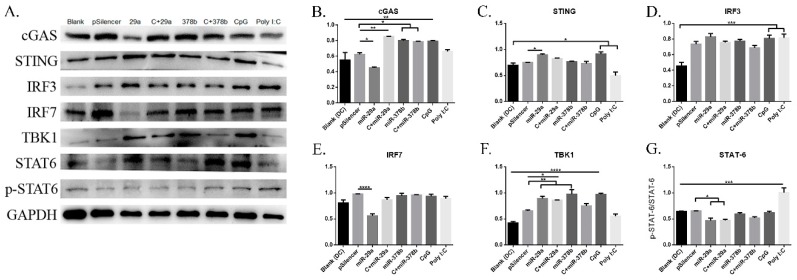
cGAS/STING pathway regulatory protein expression on BMDCs stimulated by miR-29a and miR-378b determined by Western blot. (**A**) Western blot results in naïve DCs and DCs stimulated by CpG with overexpressed miR-29a and miR-378b for the total protein level of *GAPDH*, *cGAS*, *STING*, *IRF3*, *IRF7*, *TBK1*, *STAT6*, and phosphorylated *STAT6* (lane 1: blank group; lane 2: control (pSilencer4.1)-stimulated group; lane 3: miR-29a-stimulated group; lane 4: CpG-added miR-29a-stimulated group; lane 5: miR-378b-stimulated group; lane 6: CpG-added miR-378b stimulated group; lane 7: CpG-stimulated group; lane 8: Poly I:C-stimulated group). (**B–G**) The protein expression level and band density in overexpression groups of miR-29a and miR-378b with *cGAS*, *STING*, *IRF3*, *IRF7*, *TBK1*, *STAT6*, and phosphorylated *STAT6*, respectively. The *cGAS*, *STING*, *IRF3*, *IRF7*, and *TBK1* expressions were normalized with *GAPDH*, while phosphorylated *STAT6* was normalized with *STAT6*. pSilencer4.1 vectors were used as a negative control, while CpG and Poly I:C were used as a positive control. The data shown are the means ± standard error from three independent experiments. The level of significance between blank and positive control groups and the group treated with pSilencer4.1 are identified by * *p* < 0.05, ** *p* < 0.01, *** *p* < 0.001, and **** *p* < 0.0001, determined by one-way ANOVA with Tukey’s multiple comparison test.

The experiment described above demonstrated that miR-29a and miR-378b could activate the cGAS/STING pathway at the mRNA level. Therefore, we continued to examine their effects on the cGAS/STING pathway at the protein level (Figure 5A) (Figure S6A,B, Supplementary Materials). We found that DCs treated with miR-29a showed significantly decreased protein levels of *cGAS* and *STAT-6* (Figure 5B,G), but increased levels of *STING* and *TBK1* (Figure 5C,F). Specifically, the protein level of *IRF7* was reduced by 50% compared to pSilencer4.1 in the miR-29a overexpression group (Figure 5E). Moreover, CpG-stimulated DCs pretreated with miR-29a showed increased expression of *cGAS* and *TBK1* (Figure 5B,F), but decreased expression of *STAT-6* (Figure 5G). We also found that miR-378b significantly enhanced protein levels of *cGAS* and *TBK1* (Figure 5B,F), consistent with the qPCR results. Finally, CpG-stimulated DCs preincubated with miR-378b showed highly elevated *cGAS* protein levels (Figure 5B). Together, these results suggest that *IRF7* could be regulated by miR-29a, while *TBK1* could be affected by miR-378b.

### 3.5. miR-29a and miR-378b Infliuence Mitogen-Activated Protein Kinase (MAPK) and TRAF6 Pathways in CpG-Stimulated BMDCs

**Figure 6:**
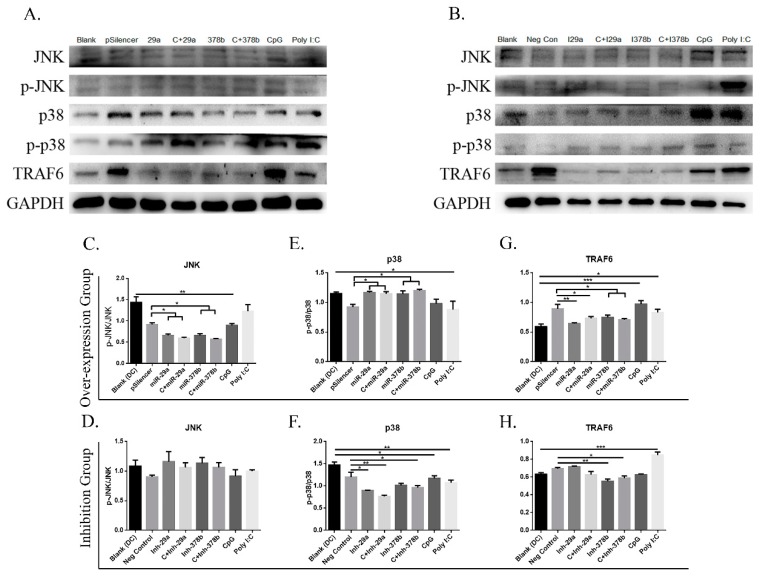
miR-29a and miR-378b influence Mitogen-Activated Protein Kinase (MAPK) and TNK Receptor Associated Factor 6 (*TRAF6*) pathways. (**A**) Cells were harvested, and Western blot analysis was performed for phosphorylation and the total level of c-Jun NH2-terminal Kinase (*JNK*) and *p38* kinases, and *TRAF6* in miRNA overexpression group; *GAPDH* protein expression was used as a loading control. (**B**) Western blot analysis of inhibited miR-29a and miR-378b on naïve DCs and CpG-stimulated DCs for phosphorylation and the total level of *JNK* and *p38* kinases, *TRAF6*, and *GAPDH* (lane 1: blank group; lane 2: control (pSilencer4.1 or negative control)-stimulated group; lane 3: miR-29a- or inhibitor miR-29a-stimulated group; lane 4: CpG-added miR-29a- or inhibitor miR-29a-stimulated group; lane 5: miR-378b- or inhibitor miR-378b-stimulated group; lane 6: CpG-added miR-378b- or inhibitor miR-378b-stimulated group; lane 7: CpG-stimulated group; lane 8: Poly I:C-stimulated group). (**C**,**D**) Western blot band density and protein expression level in overexpression and inhibitor groups of miR-29a and miR-378b with *JNK* normalized with phosphorylated *JNK*, and (**E**,**F**) *p38* normalized with phosphorylated *p38*. (**G**,**H**) Band density and protein expression level of *TRAF6* normalized with *GAPDH* in miRNA overexpression and inhibited groups. Vectors (pSilencer4.1) were used as a negative control; CpG and Poly I:C were used as a positive control. All results are taken from three independent experiments. The level of significance between blank and positive control groups and the group treated with pSilencer4.1 are identified as * *p* < 0.05, ** *p* < 0.01, *** *p* < 0.001, and **** *p* < 0.0001, determined by one-way ANOVA with Tukey’s multiple comparison test.

MAPKs are generally expressed in all cell types and play important roles in regulating gene expression and immunological responses [28,29]. To explore the mechanisms underlying CpG induction of miRNAs in BMDCs, we examined the effect of CpG on the activities of MAPKs and *TRAF6*, and their involvement in miRNA expression in DCs (Figure 6A,B). Firstly, we found that DCs incubated with miR-29a/miR-378b and CpG-stimulated DCs preincubated with miR-29a/miR-378b overexpression groups showed significantly decreased protein levels of *p-JNK/JNK*, while no change was observed after inhibition (Figure 6C,D). Secondly, the results showed that DCs treated with miR-29a/miR-378b and CpG-stimulated DCs preincubated with miR-29a/miR-378b overexpression groups had significantly increased protein levels of *p-p38/p38*, which was reduced in the corresponding inhibition groups (Figure 6E,F). Thirdly, we investigated *TRAF6* expression at both the mRNA and protein levels. The results suggested that the protein level of *TRAF-6* was downregulated in all overexpression groups (Figure 6G). Interestingly, the mRNA levels of miR-29a and miR-378b are associated with increased *TRAF6* after CpG stimulation (Figure S7A, Supplementary Materials). Furthermore, NF-κB essential modulator (*NEMO*), which is an adapter in the nuclear factor kappa-light-chain-enhancer of activated B cells (*NF-κB*) and IRF signaling pathways [30], and which connects the IκB kinase (*IKK*) complex with *TBK1* [31], showed no significant changes in mRNA level in the overexpression or inhibition groups (Figure S7A,B, Supplementary Materials).

### 3.6. CpG-Induced miRNAs Influence Cytokine Secretion by DCs

**Figure 7:**
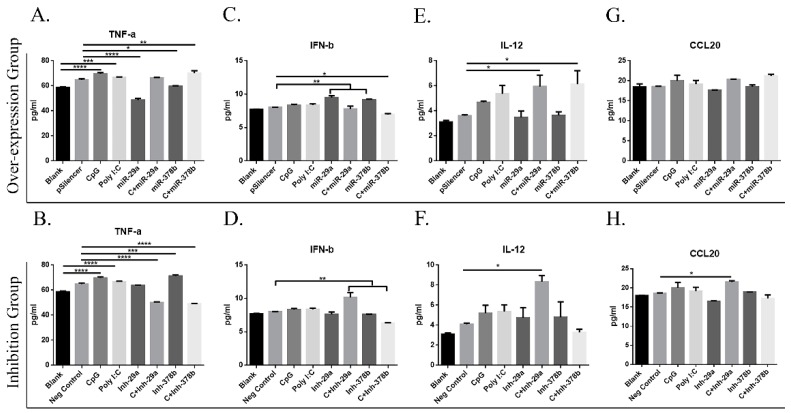
Effect of miR-29a and miR-378b on cytokine and interleukin production of BMDCs stimulated by CpG. (**A**) Tumor Necrosis Factor-α (TNF-α) overexpression group, (**B**) TNF-α inhibition group, (**C**) Interferon-β (IFN-β) overexpression group, (**D**) IFN-β inhibition group, (**E**) Interleukin-12 (IL-12) overexpression group, (**F**) IL-12 inhibition group, (**G**) C-C motif chemokine Ligand 20 (CCL20) overexpression group, and (**H**) CCL20 inhibition group. Cytokines released from naïve and CpG-stimulated BMDCs influenced by overexpressed and inhibited groups of miR-29a and miR-378b were measured by enzyme-linked immunosorbent assays. Data for TNF-α, IFN-β, IL-12, and CCL20 are shown as means ± standard deviation (SD) of three independent experiments. Significant differences between blank and positive control groups, and negative control and experimental groups are expressed as * *p* < 0.05 or ** *p* < 0.01, *** *p* < 0.001, and **** *p* < 0.0001, respectively. The significance of the data was determined by one-way ANOVA with Tukey’s multiple comparison test.

Recent studies revealed that CpG can directly induce DC maturation by promoting cytokine secretion [10]. Cell supernatants were harvested, and cytokine levels were determined to assess the ability of DCs to activate lymphocytes and secrete cytokines. The results showed that miR-29a and miR-378b significantly inhibit TNF-α production in the overexpression group (Figure 7A). IFN-β production was significantly enhanced with miR-29a and miR-378b in the overexpression group (Figure 7C), while no significant change was observed after inhibition (Figure 7D). Interestingly, CpG-stimulated DCs pretreated with an inhibitor of miR-29a showed enhanced IFN-β production (Figure 7D). The chemokine CCL20 plays an important role in the cGAS/STING pathway, but the expression of CCL20 is studied primarily in lymphocytes. Therefore, we examined whether CCL20 is exposed in DCs. The results showed no change in IL-12 or CCL20 production by DCs (Figure 7E–H). Furthermore, CpG-stimulated DCs preincubated with an inhibitor of miR-378b showed dramatic changes compared to the overexpression group in IL-12 and TNF-α production. Together, these results show that miRNAs stimulated by CpG can influence cytokine production by DCs and that miR-378b enhances CpG activity in cytokine production.

### 3.7. miR-29a and miR-378b Target Prediction and Validation

**Figure 8:**
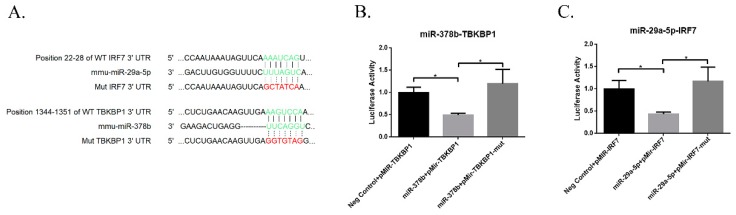
Results of dual luciferase reporter assay of miRNAs and their target genes. (**A**) Prediction of wild-type and mutant-type sequences of *IRF7* and *TBKBP1* genes with their binding sites for miR-29a-5p and miR-378b, respectively. (**B**) Dual luciferase assay results of miR-29a-5p and its target wild-type and mutant *IRF7* genes, and (**C**) miR-378b and wild-type and mutant *TBKBP1* genes. Independent miRNA was used as a negative control, transfected with the wild-type gene. Significant differences between the negative control group and miRNA target group are expressed as * *p* < 0.05, determined by one-way ANOVA with Tukey’s multiple comparison test.

Numerous studies showed that miRNAs bind to the 3′ untranslated region and regulate gene expression by degrading mRNA or interfering with protein translation. To identify the potential targets of miR-29a and miR-378b, we performed a search using TargetScan and miRDB software to predict target genes of miR-29a and miR-378b (Figure S1, Supplementary Materials). Consistent with our qPCR and Western blot results, we found that miR-29a-5p binds IRF7, while miR-378b binds *TBKBP1* (Figure 8A). We performed dual luciferase reporter assays to confirm the target site prediction for these miRNAs. The results showed that *TBKBP1* and *IRF7* significantly reduced luciferase activity compared to the negative control group, whereas *TBKBP1* and *IRF7* significantly reduced luciferase activity when the binding sites were mutated (Figure 8B,C).

## 4. Discussion

The cGAS/STING signaling pathway plays a pivotal role in cytosolic DNA sensing and elicits strong activation of DCs in the immune response [12,32]. CpG is a powerful tool for regulating the maturation of DCs, boosting the immune response against a wide variety of pathogens, enhancing cancer and allergy therapies, and developing prophylactic or therapeutic vaccines [6,11]. miRNAs are responsible for dynamic changes in gene expression and regulate innate antiviral responses [13,17]. In this study, we focused on the effects of miR-29a and miR-378b on naïve or CpG-stimulated BMDCs in term of phenotypic changes and cytokine production, as well as the cGAS/STING and MAPK/TRAF6 pathways.

Accumulating evidence revealed that miRNAs inhibit key regulatory components of the innate immune response and markedly affect the capacity of DCs to undergo phenotypic changes [22,23,33,34]. Our findings suggest that both miR-29a and miR-378b could enhance DC maturation by increasing surface marker expression of CD40 and CD86 in the naïve condition. However, when BMDCs were stimulated with CpG, the results differed. miR-378b treatment did not affect the surface markers (MHCII and CD80) when DCs were stimulated with CpG. On other hand, CD273 and Siglec-G are immunosuppressive molecules. Siglec-G inhibits DC cross-presentation and suppresses the innate immune response [2,35,36]. CD273 binds to the programmed cell death protein-1 (PD-1) receptor on T-cells and causes downregulation of various functions, including T-cell proliferation and cytokine production [37]. Consistent with Abomaray [26], we found that miR-29a and miR-378b significantly increased the expression of CD273, but significantly reduced the expression of Siglec-G molecules in BMDCs. When miR-378b was inhibited, CD273 decreased significantly. Together, our results suggest that miR-378b and miR-29a might be inhibitory miRNAs.

To further demonstrate the inhibitory function of miR-378b, we detected the cytokine production of TNF-α, IFN-β, IL-12, and CCL20. Ma found that miRNA-29 family members control the immune system and host–pathogen interactions and influence cytokine (IFN, TNF, and interleukin) signaling in response to bacterial and viral infections [38]. The miR-378 family was observed to influence cell proliferation, and to suppress tumour growth and angiogenesis through various signaling pathways and cytokine production patterns [39,40,41,42,43,44,45]. Our findings show that miR-29a increases IFN-β production and decreases the expression of TNF-α. When miR-29a was inhibited, combined with CpG treatment, a significant decrease in TNF-α and a significant increase in IFN-β production were observed compared with the CpG group without inhibition. miR-378b reduced the expression of the pro-inflammatory cytokine TNF-α and increased IFN-β production. Thus, miR-29a and miR-378b regulate cytokine production by DCs, and miR-378b has an inhibitory role, suppressing the stimulatory effect of CpG on activating cytokine secretion.

Recent findings showed that CpG-stimulated DCs induce the MAPK [18,46] and cGAS/STING signaling pathways [12,47]. cGAS/STING is an essential cytosolic DNA sensing pathway that drives the induction of the DNA-mediated immune response, irrespective of cell type or DNA sequence [48,49,50]. Recently, *cGAS* and *STING* were identified as intracellular sensors that activate the interferon pathway and mediate host defense; this role might be influenced by miRNAs [12,32,49]. Wu showed that miR25/93 mediates hypoxia-induced immunosuppression by repressing *cGAS* [51], and Huang revealed that miR-24 regulates *STING* in rats [15]. Similarly, our results showed that miR-378b could bind *TBKBP1* and increase the expression of *TBK1* to activate the cGAS/STING pathway. Unlike miR-378b, we found that miR-29a could bind *IRF7* and activate STING/TBK1/IRF3. Moreover, miR-29a could downregulate *TRAF6* expression, which might be involved in reducing the antiviral response, consistent with research by Carrie [52]. In addition, Chunyan found that *JNK* and *p-38* kinases are involved in CpG-induced CD40 expression in BMDCs [46]. Nagalingam showed that miR-378 reduces the activity of MAPK [53]. Consistent with that result, our research showed that both miR-29a/miR-378b and CpG-stimulated miR-29a/miR-378b groups had significantly elevated p-p38/p38 signaling and decreased *p-JNK/JNK* signaling.

## 5. Conclusions

In summary, we describe an interaction between miRNAs and CpG, promoting the activation of BMDCs. Firstly, we selected six miRNAs whose expression levels were altered by CpG in BMDCs. Secondly, we found that the interaction of miR-29a and miR-378b substantially regulates the maturation of DCs by influencing immune function, including through phenotypic alteration and cytokine production. Thirdly, we demonstrated that miR-378b could target *TBKBP1* to activate the cGAS/STING pathway, while miR-29a binds to *IRF7* to inhibit IRF7/TRAF6. These findings elucidate the role of miRNAs in the regulation of CpG-stimulated DC maturation, which may accelerate studies on immune response and vaccine production. We present a previously uncharacterized mechanism for miRNA-mediated restriction of the cytosolic DNA sensing pathway.

## Figures and Tables

**Table 1:** qRT-PCR primers used in verification of microRNA (miRNA) results.

miRNAs	Sense Primers (5′ to 3′)
mmu-miR-29a	AGAGGATGACTGATTTCTTTTGGTGTTC
mmu-miR-98	GCTGGGGTGAGGTAGTAAGTTGTATTGT
mmu-miR-196a-2	GTGGCTTAGGTAGTTTCATGTTGTTGG
mmu-miR-222	AGTGGCTCAGTAGCCAGTGTAGATCC
mmu-miR-361	GAAGCTTATCAGAATCTCCAGGGGTAC
mmu-miR-378b	GGGAACCCTGGACTTGGAGTCAGAAGA

**Table 2:** Primers used in amplification of mouse miRNAs.

miRNAs	Sequence	Products
miR-29a sense	GGCTCGAGACACCCACCATCACTATGTG	461 bp
miR-29a anti-sense	TGAATTCAACAGGCTACCAGAGCCTG	
miR-378b sense	GGCTCGAGTATCATGGCATATCAGCAGAAGC	488 bp
miR-378b anti-sense	TGAATTCTGCATATACCTGATAAGTCACAGG	

**Table 3:** Primers used in amplified mmu-miRNA target gene.

Gene	Sequence	Products
mmu-miR-29a-5p target gene-Interferon Regulatory Factor 7 (*IRF7*) wild-type sense	AAGATCCTTTATTAAGCTTAAATCAGTGGAGCCCTGGGT	**406 bp**
mmu-miR-29a-5p target gene-*IRF7* wild-type anti-sense	GCACTAGTGAGGGAGCTCGGTTTCGGAAAGCCTGACGG	
mmu-miR-29a-5p target gene-*IRF7* mutant-type sense	AAGATCCTTTATTAAGCTTGCTATCATGGAGCCCTGGGT	
mmu-miR-378b target gene-TANK-Binding Kinase Binding Protein 1 (*TBKBP1*) wild-type sense	GATCCTTTAAGCTTAAGTCCAATAAAACTTACCTG	**483 bp**
mmu-miR-378b target gene-*TBKBP1* wild-type anti-sense	CACTAGTGAGGGAGCTCAAAAGTCATGAGTTTGTGAC	
mmu-miR-378b target gene-*TBKBP1* mutant type sense	GATCCTTTATTAAGCTTGGTGTAGGATAAAACTTACCTG	

**Table 4:** qRT-PCR primers used in verification of messenger RNA (mRNA) results.

Gene	Sense	Anti-Sense
***GAPDH***	AGGTCGGTGTGAACGGATTTG	GGGGTCGTTGATGGCAACA
***cGAS***	CATGTGTGCAGGAGCATGTA	CAACAACCCATGCAACAAAG
***STING***	TGAGCCTCAACCAACCCTAC	CCATCCACACAGGTCAACAG
***TBK1***	GGGGTGCTCTCCCTAATTCT	CTTGTCAGGGAACCGACTGT
***TBKBP1***	GGTGAGGGGTATGTCAGCAG	TCCAAGACCTTCCGAGTGAG
***IRF3***	CAAGGCTCAGTCTTCCCATC	CGTAGGGACAATGTGTGTGC
***IRF7***	TCTCGGCTTGTGCTTGTCTA	ACTGGGGGTCACCTTCTTTC
***STAT6***	TCTGCTTTTGCCAGTGTGAC	GCCCAGGGAGTTTACACAGA
***TRAF6***	TGGGTCCTCTGTGTCTTTGA	AGGAGTCAGATGGGGCTACA
***NEMO***	GCTCCTGTTGTCTCCTTTGC	TGCAGTTTCCCTGTGTTGAG

## Supplementary Materials

The following are available online at https://www.mdpi.com/2076-393X/7/4/197/s1: Figure S1: The Venn diagram showing the miRNA–mRNA interaction predicted from two different software, TargetScan and miRDB in common: (A) mmu-miR-29a-5p target site *IRF7* prediction from TargetScan and miRDB in common (B) mmu-miR-378b target site *TBKBP1* prediction from TargetScan and miRDB in common; Figure S2: Identification and construction of pSilencier4.1-mmu-miR-29a and pSilencier4.1-mmu-miR-378b by digestion of *BamH*I and *Hind*III. (A) Identification of constructed pSilencier4.1-mmu-miR-29a by digesting with *BamH*I and *Hind*III (M: DL10,000 DNA Marker, 1 and 2: mmu-miR-29a and Plasmid pSilencier4.1 digested with *BamH*I and *Hind*III). (B) Identification of constructed pSilencier4.1-mmu-miR-378b by digesting with *BamH*I and *Hind*III (M: DL10,000 DNA Marker, 1 and 2: mmu-miR-378b and Plasmid pSilencier4.1 digested with *BamH*I and *Hind*III); Figure S3: Identification and construction of pMIR-Report vector-*IRF7*, and pMIR-Report vector-*TBKBP1* by digesting *Hind*III and *Sac*I. (A) Identification of constructed pMIR-Report vector-*IRF7*-wild type digesting by *Hind*III and *Sac*I (M: DL10,000 DNA Marker, 1 and 2: amplified *IRF7* wild-type gene and pMIR-Report vector digested with *Hind*III and *Sac*I). (B) Identification of constructed pMIR-Report vector-*IRF7*-mutant digestion by *Hind*III and *Sac*I (M: DL10,000 DNA Marker, 1 and 2: amplified *IRF7* mutant and pMIR-Report vector digested with *Hind*III and *Sac*I). (C) Identification of constructed pMIR-Report vector-*TBKBP1*-wild type digesting by *Hind*III and *Sac*I (M: DL5,000 DNA Marker, 1: amplified *TBKBP1* wild-type gene and pMIR-Report vector digested with *Hind*III and *Sac*I). (D) Identification of constructed pMIR-Report vector-*TBKBP1*-mutant digested by *Hind*III and *Sac*I (M: DL5,000 DNA Marker, 1: amplified *TBKBP1* mutant and pMIR-Report vector digested with *Hind*III and *Sac*I); Figure S4: Phenotypic alterations of mouse immature BMDCs in response to CpG and miRNAs. Morphological observation of BMDCs stimulated by GM-CSF and IL-4 for seven days (line 1: only DCs (blank sample), line 2: DCs transfected with pSilencer4.1 and negative control for miRNAs, line 3: DCs stimulated with CpG and Poly I:C, line 4: DCs transfected with miR-29a and inhibitor of miR-29a, line 5: CpG-stimulated DCs transfected with miR-29a and inhibitor of miR-29a, line 6: immature DCs transfected with miR-378b and inhibitor of miR-378b, line 7: CpG-stimulated DCs transfected with miR-378b and inhibitor of miR-378b); Figure S5: Results of qPCR analysis following stimulation by inhibited miR-29a and miR-378b of cGAS/STING pathway related genes: *cGAS*, *STING*, *TBK1*, *IRF3*, *IRF7*, *TBKBP1*, and *STAT6*. All these expressions were normalized with *GAPDH* mRNA expression level. These results are taken from three independent experiments. Significant differences between the blank and positive control groups and group treated by pSilencer4.1 are expressed as * *p* < 0.05, ** *p* < 0.01, *** *p* < 0.001, and **** *p* < 0.0001, determined by one-way ANOVA with Tukey’s multiple comparison test; Figure S6: cGAS/STING pathway regulatory protein expression on BMDCs stimulated by miR-29a and miR-378b determined by Western blot. (A) Western blot results in naïve DCs and DCs stimulated by CpG with inhibited miR-29a and miR-378b for the total protein level of *GAPDH*, *cGAS*, *STING*, *IRF3*, *IRF7*, *TBK1*, *STAT6*, and phosphorylated *STAT6* (lane 1: blank group; lane 2: control (pSilencer4.1)-stimulated group; lane 3: miR-29a-stimulated group; lane 4: CpG-added miR-29a-stimulated group; lane 5: miR-378b-stimulated group; lane 6: CpG-added miR-378b-stimulated group; lane 7: CpG-stimulated group; lane 8: Poly I:C-stimulated group). (B) The protein level and band density in overexpression groups of miR-29a and miR-378b with *cGAS*, *STING*, *IRF3*, *IRF7*, *TBK1*, *STAT6*, and phosphorylated *STAT6*, respectively. The data shown are the means ± standard error from three independent experiments. The level of significance between blank and positive control groups and group treated by pSilencer4.1 are identified by * *p* < 0.05, ** *p* < 0.01, *** *p* < 0.001, and **** *p* < 0.0001, determined by one-way ANOVA with Tukey’s multiple comparison test; Figure S7: qPCR analysis of mRNA expression of *TRAF6* and *NEMO* pathway. (A) qPCR analysis following stimulation by over-expressed miR-29a and miR-378b of *TRAF6* and *NEMO*; (B) qPCR analysis following stimulation by inhibited miR-29a and miR-378b of *TRAF6* and *NEMO*. qPCR mRNA expressions of *TRAF6* and *NEMO* were normalized with mRNA expression of *GAPDH*. These results are taken from three independent experiments. Significant differences between blank and positive control groups and group treated by pSilencer4.1 are expressed as * *p* < 0.05, ** *p* < 0.01, *** *p* < 0.001, and **** *p* < 0.0001, determined by one-way ANOVA with Tukey’s multiple comparison test; Table S1: Key resources tables include the list of all reagents, source and identifier of FACS antibodies, WB antibodies, vectors and inhibitors, chemicals, peptides and recombinant proteins, critical commercial assays and software used in this study.

## Abbreviations

DCs	dendritic cells
miRNA	microRNA
CpG	Cytosine–phosphate–guanosine
BMDC	bone marrow dendritic cells
GM-CSF	granulocyte-macrophage colony-stimulating factor
MHCII	major histocompatibility complex class II
Siglec-G	sialic acid-binding immunoglobulin-type lectin-G
3′UTR	three-prime untranslated region
FACS	Fluorescence-Activated Cell Sorter
MAPK	mitogen-activated protein kinases
cGAS/STING	cyclic GMP–AMP/synthase-stimulator of interferon genes
*TBKBP1*	TANK-binding kinase binding protein 1
*IRF7*	interferon regulatory factor 7
TLR-9	Toll-like receptor 9
