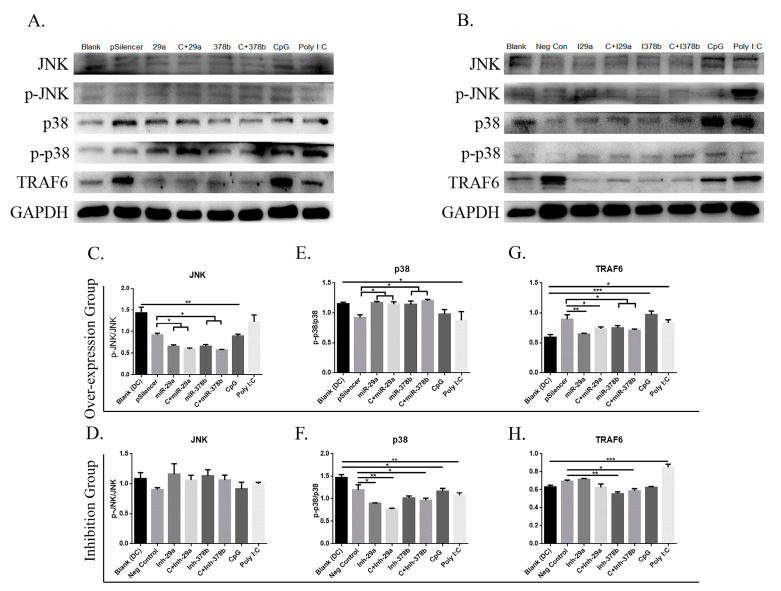# Correction: miR29a and miR378b Influence CpG-Stimulated Dendritic Cells and Regulate cGAS/STING Pathway

**DOI:** 10.3390/vaccines8020305

**Published:** 2020-06-16

**Authors:** Abid Ullah Shah, Yanan Cao, Naila Siddique, Jian Lin, Qian Yang

**Affiliations:** 1 College of Veterinary medicine, Nanjing Agricultural University, Wei gang 1, Nanjing 210095, China; abidullahshah@yahoo.com (A.U.S.); yncao1994@163.com (Y.C.); 2 National Reference Laboratory for Poultry Diseases, Animal Sciences Institute, National Agricultural Research Center, Islamabad 44000, Pakistan; naila.nrlpd@gmail.com; 3 College of Life Sciences, Nanjing Agricultural University, Wei gang 1, Nanjing 210095, China

The authors wish to make the following corrections to this paper [1]: 

The authors would like to apologize for any inconvenience caused to the readers by these changes.

The authors wish to make the following correction to this paper [1]: Due to our mishandling we inserted a wrong image of GAPDH in Figure 6B; therefore, we want to replace the GAPDH image of Figure 6B with the old one:


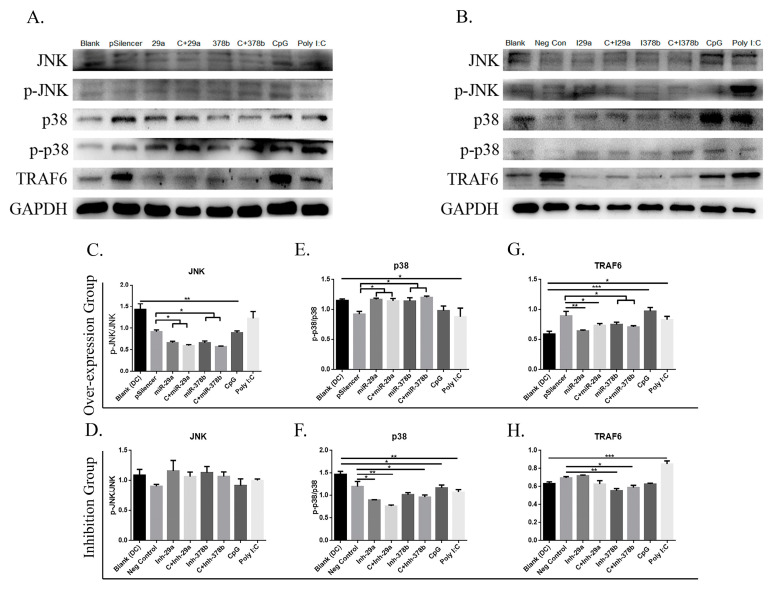
 with