# Supplementary material for: miR29a and miR378b Influence CpG-Stimulated Dendritic Cells and Regulate cGAS/STING Pathway
Source: Vaccines (Basel). 2019 Nov 26;7(4):197. doi: 10.3390/vaccines7040197 (PMC6963666; doi:10.3390/vaccines7040197)
Supplement: Supplementary file 1 [file vaccines-07-00197-s001.zip › vaccines-604607 author confirm sup.pdf]

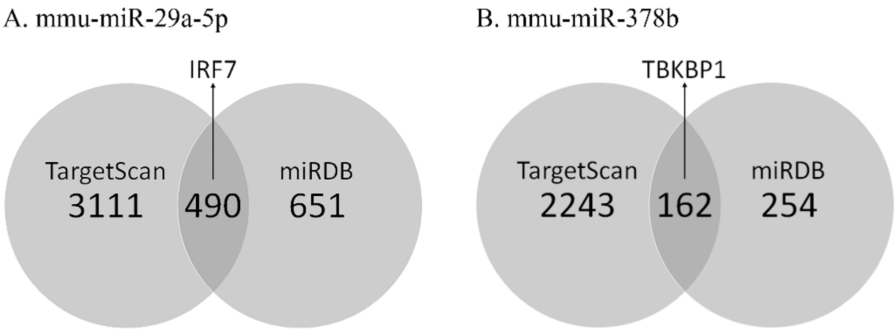


**Figure S1:** The Venn diagram showing the miRNA-mRNA interaction predicted from two different software, TargetScan and miRDB in common (**A)** mmu-miR-29a-5p target site *IRF7* prediction from TargetScan and miRDB in common **(B)** mmu-miR-378b target site *TBKBP1* prediction from TargetScan and miRDB in common.

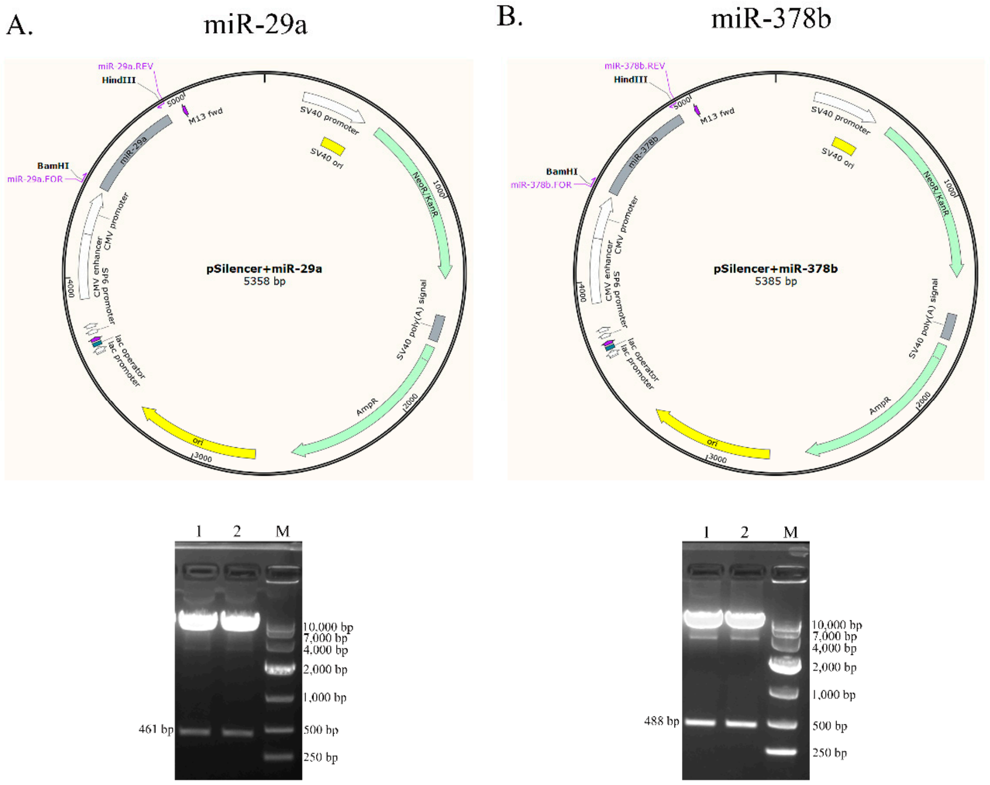


**Figure S2:** Identification and construction of pSilencier4.1-mmu-miR-29a and pSilencier4.1-mmu-miR-378b by digestion of BamHI and Hind III. (**A)** Identification of constructed pSilencier4.1-mmu-miR-29a by digesting with BamHI and Hind III (M: DL10,000 DNA Marker, 1 and 2: mmu-miR-29a and Plasmid pSilencier4.1 digested with BamHI and Hind III). **(B)** Identification of constructed pSilencier4.1-mmu-miR-378b by digesting with BamHI and Hind III (M: DL10,000 DNA Marker, 1 and 2: mmu-miR-378b and Plasmid pSilencier4.1 digested with BamHI and Hind III).

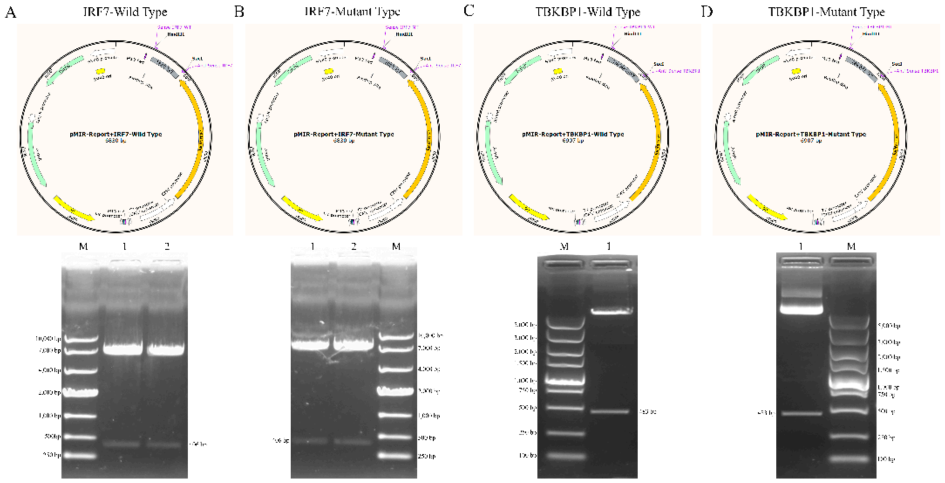


**Figure S3:** Identification and construction of pMIR-Report vector-*IRF7*, and pMIR-Report vector-*TBKBP1* by digesting Hind III and Sac I. **(A)** Identification of constructed pMIR-Report vector-*IRF7*-Wild Type digesting by Hind III and Sac I (M: DL10,000 DNA Marker, 1 and 2: amplified *IRF7* wild type gene and pMIR-Report vector digested with Hind III and Sac I). **(B)** Identification of constructed pMIR-Report vector-*IRF7*-Mutant digestion by Hind III and Sac I (M: DL10,000 DNA Marker, 1 and 2: amplified *IRF7* mutant and pMIR-Report vector digested with Hind III and Sac I). **(C)** Identification of constructed pMIR-Report vector-*TBKBP1*-Wild Type digesting by Hind III and Sac I (M: DL5,000 DNA Marker, 1: amplified *TBKBP1* wild type gene and pMIR-Report vector digested with Hind III and Sac I). **(D)** Identification of constructed pMIR-Report vector-*TBKBP1*-Mutant digested by Hind III and Sac I (M: DL5,000 DNA Marker, 1: amplified *TBKBP1* mutant and pMIR-Report vector digested with Hind III and Sac I).

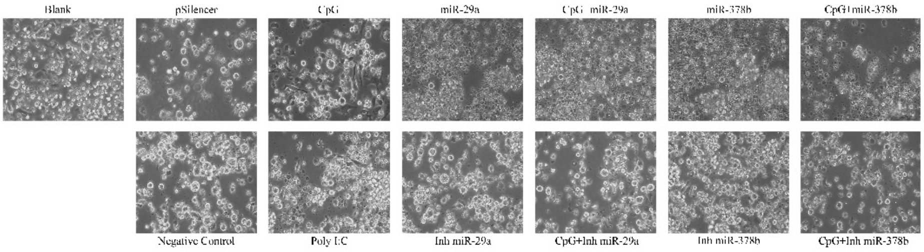


**Figure S4:** Phenotypic alterations of mouse immature BMDCs in response to CpG and miRNAs. Morphological observation of BMDCs stimulated by GM-CSF and IL-4 for 7 days. (Line 1: only DCs (Blank sample), Line 2: DCs transfected with pSilencer4.1 and negative control for miRNAs, Line 3: DCs stimulated with CpG and Poly I:C, Line 4: DCs transfected with miR-29a and inhibitor of miR-29a, Line 5: CpG-stimulated DCs transfected with miR-29a and inhibitor of miR-29a, Line 6: Immature DCs transfected with miR-378b and inhibitor of miR-378b, Line 7: CpG-stimulated DCs transfected with miR-378b and inhibitor of miR-378b).

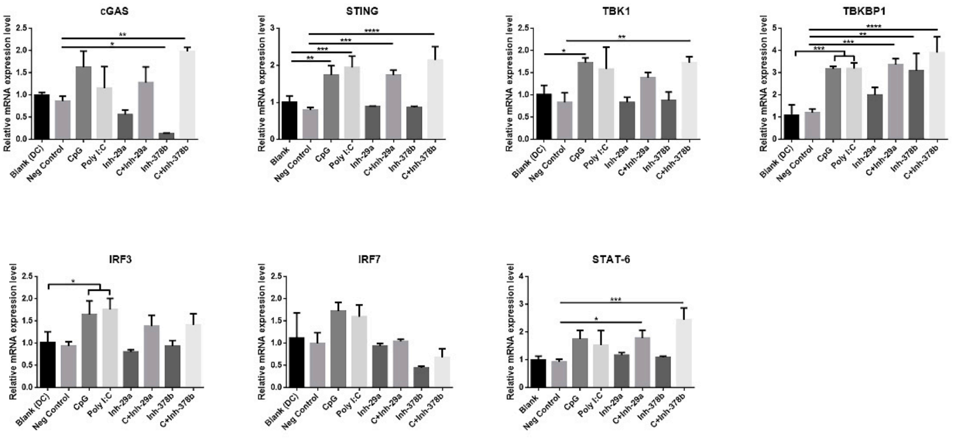


**Figure S5:** Results of qPCR analysis following stimulation by inhibited miR-29a and miR-378b of cGAS/STING pathway related genes. *cGAS*, *STING*, *TBK1*, *IRF3*, *IRF7*, *TBKBP1* and *STAT6*. All these expressions were normalized with *GAPDH* mRNA expression level. These results are taken from three independent experiments. Significant differences between the Blank with positive control groups, and treated with pSilencer4.1 groups are expressed as *P < 0.05, **P < 0.01, ***P < 0.001 and ****P < 0.0001, determined by one-way ANOVA with Tukey's multiple comparison test.

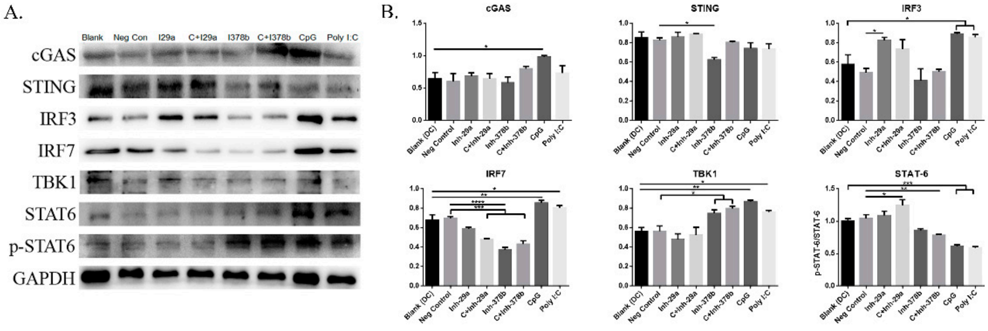


**Figure S6:** cGAS/STING pathway regulatory protein expression on BMDCs stimulated by miR-29a and miR-378b determined by western blot. **(A)** Western blot results in naïve DCs and DCs stimulated by CpG with inhibited miR-29a and miR-378b for the total protein level of *GAPDH*, *cGAS*, *STING*, *IRF3*, *IRF7*, *TBK1*, *STAT6* and phosphorylated *STAT6*. (lane 1: blank group; lane 2: control (pSilencer4.1) stimulated group; lane 3: miR-29a stimulated group; lane 4: CpG added miR-29a stimulated group; lane 5: miR-378b stimulated group; lane 6: CpG added miR-378b stimulated group; lane 7: CpG stimulated group; lane 8: Poly I:C stimulated group.) **(B)** The protein level and band density in over-expression groups of miR-29a and miR-378b with *cGAS*, *STING*, *IRF3*, *IRF7*, *TBK1*, *STAT6* and phosphorylated *STAT6* respectively. The data shown are the means ± standard error from three independent experiments. The level of significance between blank with positive control groups, and treated with pSilencer4.1 group are identified by *$P$ < 0.05, **$P$ < 0.01, ***$P$ < 0.001 and ****P < 0.0001, determined by one-way ANOVA with Tukey's multiple comparison test.

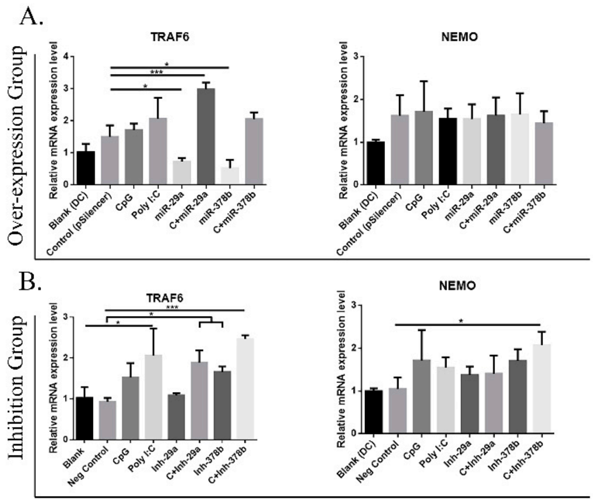


**Figure S7:** qPCR analysis of mRNA expression of *TRAF6* and *NEMO* pathway. **(A)** qPCR analysis following stimulation by over-expressed miR-29a and miR-378b of *TRAF6* and *NEMO*, **(B)** qPCR analysis following stimulation by inhibited miR-29a and miR-378b of *TRAF6* and *NEMO*. qPCR mRNA expression of TRAF6 and NEMO were normalized with mRNA expression of *GAPDH*. These results are taken from three independent experiments. Significant differences between the Blank with positive control groups, and treated with pSilencer4.1 groups are expressed as *P < 0.05, **P < 0.01, ***P < 0.001 and ****P < 0.0001, determined by one-way ANOVA with Tukey's multiple comparison test.
