# Supplementary material for: miR29a and miR378b Influence CpG-Stimulated Dendritic Cells and Regulate cGAS/STING Pathway
Source: Vaccines (Basel). 2019 Nov 26;7(4):197. doi: 10.3390/vaccines7040197 (PMC6963666; doi:10.3390/vaccines7040197)
Supplement: Supplementary file 1 [file vaccines-07-00197-s001.zip › vaccines-604607 Table S1.pdf]

Table S1. Key Resources

Reagent or Resources	Source	Identifier
**FACS Antibodies**		
**APC-conjugated monoclonal anti-mouse CD11c**	eBioscience	REF: 17-0114-81
**PE-conjugated monoclonal anti-mouse CD40 (Clone: 1C10)**	eBioscience	REF: 12-0401-81
**PE-conjugated monoclonal anti-mouse CD80 (Clone: 16-10A1)**	eBioscience	REF: 12-0801-81
**PE-conjugated monoclonal anti-mouse CD86 (Clone: GL1)**	eBioscience	REF: 12-0862-82
**APC-conjugated monoclonal anti-mouse MHC class II (I-A/I-E) Clone: M5/114.15.2**	eBioscience	REF: 17-5321-81
**APC-conjugated monoclonal anti-mouse CD273 (B7-DC, PD-L2) Clone: TY25**	eBioscience	Cat: 107210
**APC-conjugated monoclonal anti-mouse Siglec-G (Clone: SH2.1)**	eBioscience	REF: 17-5833-80

**WB Antibodies**		
cGAS **(*MB21D1*)**	Bioworld	BS71168
STING **(*TMEM173*)**	Bioworld	BS70551
TBK1	Bioworld	BS60714
STAT6 **(R639)**	Bioworld	BS1341
p-STAT6 **(phospho-Y641)**	Bioworld	BS4187
JNK **(P184)**	Bioworld	BS1544
p-JNK **(phosphor-T183/Y185)**	Bioworld	BS4322
p38 **(T175)**	Bioworld	BS3567
p-p38 **(phosphor-Y182)**	Bioworld	BS4766
GAPDH	Bioworld	AP0063
IRF3 **(EPR2418Y)**	abcam	AB68481
IRF7 **(EPR4718)**	abcam	AB109255
TRAF6	abcam	AB137452

**Vectors and Inhibitors**		
**pSilencer4.1 overexpression vector**	Invitrogen	N/A
**pMIR-Report luciferase vector**	Ambion	N/A
**pRL-TK luciferase vector**	Ambion	N/A
**micrOFFTM mmu-miR-29a-5p inhibitor**	RIBOBIO	miR20004718
**micrOFFTM mmu-miR-378b inhibitor**	RIBOBIO	miR212824104115
**micrOFFTM inhibitor Negative Control**	RIBOBIO	miR02101

**Chemicals, Peptides and Recombinant Proteins**		
**CpG oligodeoxynucleotides mouse (1018)**	Novus Biologicals	NBP2-31142
**Poly I:C**	Merck	N/A
**Recombinant murine granulocyte-macrophage colony-stimulating factor (GM-CSF)**	Peprotech	Cat # 214-14-204G
**Recombinant murine IL-4**	Peprotech	Cat # 315-03-204G
**X-tremeGENE HP DNA Transfection Reagent**	Roche	REF: 06366546001
**Lipofectamine2000**	Invitrogen	REF: 11668-027
**5S rRNA internal control for qPCR**	RIBOBIO	N/A
**Bam HI**	BioLabs New England	N/A
**Hind III**	BioLabs New England	N/A
**Sac I**	BioLabs New England	N/A

**Critical Commercial Assays**		
**miDETECT A Track miRNA qRT-PCR Starter kit**	RIBOBIO	Cat # C10712-1
**miScript Reverse Transcriptase**	Bio-Rad	N/A
**QuantiTect SYBR Green PCR master mix**	Qiagen	N/A
**Dual-luciferase reporter assay kit**	Promega	REF: E1910
**Mouse TNF-α Elisa Kit**	SenBeiJia Biological	REF: SBJ-M0030-96T
**Mouse IL-12 Elisa Kit**	SenBeiJia Biological	REF: SBJ-M0594-96T
**Mouse IFN-β Elisa Kit**	SenBeiJia Biological	REF: SBJ-M0047-96T
**Mouse MIP-3α/CCL20 Elisa Kit**	SenBeiJia Biological	REF: SBJ-M0887-96T

**Software**		
**FlowJo V10 software**	FlowJo Software	N/A
**ImageJ**	ImageJ Software	N/A
**GraphPad Prism v6.01**	GraphPad Software	N/A
